# Expression patterns of intestinal calcium transport factors and ex-vivo absorption of calcium in horses

**DOI:** 10.1186/1746-6148-7-65

**Published:** 2011-10-22

**Authors:** Nele Sprekeler, Tobias Müller, Mariusz P Kowalewski, Annette Liesegang, Alois Boos

**Affiliations:** 1 Institute of Veterinary Anatomy, Vetsuisse Faculty, University of Zurich, Winterthurerstrasse 260, 8057 Zurich, Switzerland; 2 Institute of Animal Nutrition, Vetsuisse Faculty, University of Zurich, Winterthurerstrasse 260, 8057 Zurich, Switzerland

## Abstract

**Background:**

In many species, the small intestine is the major site of calcium (Ca^2+^) absorption. The horse differs considerably from most other species with regard to the physiology of its Ca^2+ ^metabolism and digestion. Thus, this study was performed to get more information about the transcellular Ca^2+ ^absorption in the horse.

Two mechanisms of intestinal Ca^2+ ^absorption are described: the passive paracellular pathway and the active, vitamin D-dependent transcellular pathway. The latter involves the following elements: vitamin D receptors (VDR), transient receptor potential vanilloid channel members 5 and 6 (TRPV5/6), calbindin-D9k (CB), the Na/Ca exchanger (NCX1) and the plasma membrane Ca-ATPase (PMCA). The aim of the present study was to investigate the protein and mRNA expression patterns of VDR, CB and TRPV6 and the ex-vivo Ca^2+ ^absorption in horses, assessed by qualitative and quantitative RT-PCR, western blot, immunohistochemistry and the Ussing chamber technique.

**Results:**

Highest CB and TRPV6 mRNA levels were detected in the duodenum as compared to the middle parts of the jejunum and ileum and several sites of the large intestine. VDR mRNA levels did not change significantly throughout the intestine. TRPV5 mRNA was not detectable in the horse intestine. The highest VDR and CB protein levels were measured in the duodenum. Ussing chamber studies revealed ex-vivo Ca^2+ ^absorption only in the duodenum, but not in cecum and specific sites of the colon.

**Conclusion:**

The present findings suggest that TRPV6, CB and VDR may be involved in active intestinal Ca^2+ ^absorption in horses, as described for other mammals. TRPV5 may not play a major role in this process. Furthermore, the expression patterns of these Ca^2+ ^transport elements and the results of the Ussing chamber procedure indicate that a significant part of active intestinal Ca^2+ ^absorption occurs in the duodenum in this species.

## Background

Calcium (Ca^2+^) is involved in many physiological processes, such as bone mineralisation, muscle contractions, neuronal excitability, blood coagulation, cell adhesion and apoptosis [1]. Intestinal Ca^2+ ^absorption is the main process to obtain Ca^2+ ^from nutrients. Two mechanisms of intestinal Ca^2+ ^absorption are: the paracellular and the transcellular pathways [2]. The paracellular pathway is a passive, nonsaturable process that is driven by an electrochemical gradient across the epithelium [3,4] and principally occurs when dietary Ca^2+ ^is abundant [5]. If dietary Ca^2+ ^is restricted or Ca^2+ ^demand is increased, the transcellular transport is the essential mechanism for Ca^2+ ^absorption from the diet [3,4]. This pathway is an active, vitamin D-dependent, saturable Ca^2+ ^transport that is based upon three steps: Ca^2+ ^enters from the luminal side into the enterocyte through the transient receptor potential vanilloid channel type 5 and 6 (TRPV5/6) [6,7]; the cytosolic Ca^2+ ^is transferred from the luminal to the basolateral membrane bound to calbindin-D9k (CB)[8,9]; the extrusion of Ca^2+ ^is performed by the plasma membrane Ca-ATPase (PMCA), supported by the Na/Ca exchanger (NCX1)[10,11]. Calcitriol or 1,25-dihydroxycalciferol, the active form of vitamin D (VD), is known to regulate the gene transcription of Ca^2+ ^transport factors TRPV6, CB, PMCA and NCX1 through its binding to the nuclear vitamin D receptor (VDR) in the enterocyte [3,12-14]. Under physiological conditions, the small intestine represents the major site of active Ca^2+ ^absorption in most species, including rats [15], dogs [16], sheep [17] and horses [18,19]; the small intestine is responsible for approximately 90% of the total Ca^2+ ^absorption, whereas the rate of absorption in the large intestine appears to be less than 10% [4,20,21]. Interestingly, rabbits absorb a considerable amount of Ca^2+ ^in the cecum [22,23]. Horses are typical hindgut fermenters, similar to rabbits or other small herbivores, and the specific gross anatomy of the gastrointestinal tract (i.e., the size of the large intestine) reflects its functional importance. In addition to the specific anatomic characteristics, horses differ from other mammals in their Ca^2+ ^physiology. In comparison with other mammals, the horse exhibits increased serum Ca^2+ ^concentrations, low mean serum calcidiol and vitamin D concentrations and high intestinal Ca^2+ ^absorption and urinary extrusions [18,19,24,25]. These characteristics lead to the question if specific mechanisms of intestinal Ca^2+ ^absorption occur in the horse. Recently, Rourke et al. [19] investigated the mRNA expression of several Ca^2+ ^transport elements in the gastrointestinal tract of horses and found that the small intestine seems to be the main site of transcellular Ca^2+ ^absorption. However, studies on intestinal protein expression and localisation of Ca^2+ ^transporters in horses are rare. This study was performed to further examine the site of intestinal Ca^2+ ^absorption in the horse. Therefore, the expression patterns of VDR, TRPV6, and CB mRNA and VDR and CB protein levels were examined by reverse transcriptase PCR, real-time PCR, western blot and immunohistochemistry. Furthermore, the intestinal ex-vivo absorption of Ca^2+ ^was measured using the Ussing chamber technique.

## Methods

### Animals and sampling procedures

Eight adult mares and three geldings, aged between 5 and 20 years, were included in the present study. The horses were clinically healthy, information about previous feeding and husbandry was lacking. The horses were slaughtered for commercial use, i.e., human consumption, at a local slaughterhouse irrespective of this project. Samples were taken from the following sites: beginning of the descending duodenum (DD), middle part of the jejunum (JE), middle part of the ileum (IL), body of the cecum (CC), right ventral part of the ascending colon (CAV), right dorsal part of the ascending colon (CAD) and terminal part of the descending colon (CD).

For the Ussing chamber experiments, the DD, CC, CAV and CD samples were collected within 10 minutes after death and placed in a Parson buffer solution, aerated with oxycarbon and immediately transported to the laboratory. The Parson buffer solution contained (in mmol l^-1^): NaCl, 107; KCl, 4.5; NaHCO_3_, 25; Na_2_HPO_4_, 1.8; NaH_2_PO_4_, 0.2; CaCl_2_, 1.25; MgSO_4_, 1; and glucose, 12.2. The solution was gassed with 5% CO_2 _in 95% O_2 _and kept at 37°C. The pH was adjusted to 7.4.

Within 20 minutes after death, samples for western blot and PCR were taken and placed in Trasylol solution (Bayer, Zürich, CH), frozen in liquid nitrogen and stored at -80° C. Tissue samples for immunohistochemistry were rinsed in 0.9% saline, fixed in 4% neutral buffered formalin for 24 hours and finally embedded in paraffin (Histowax, Leica) as previously described [26].

### RNA isolation and reverse transcriptase

Total RNA was isolated from all intestinal segments using TRIzol Reagent (Invitrogen) according to the manufacturer's protocol. The amount of RNA was quantified by spectrophotometry (SmartSpec™ Plus Spectrophotometer, BIO RAD). Purity and quality of mRNA was determined by optical density (OD) measurement. The OD 260/280 ratio of all samples was greater than 1.8. Then, 200 ng of total RNA was treated with DNase to eliminate genomic DNA contamination (DNase I recombinant, RNase free, Roche Diagnostics, Indianapolis, IN) and subjected to reverse transcription using the Gold RNA PCR Core Kit (Gene Amp). The RT reaction was run for 8 minutes at 21°C, 15 minutes at 45°C and 5 minutes at 99°C.

### Qualitative RT-PCR

Complementary DNA (cDNA) was amplified under the following hot-start PCR conditions: activation of Amplitaq Gold DNA polymerase at 95° C for 10 minutes, followed by 40 cycles of denaturation at 94°C for one minute, annealing at 60°C for two minutes, extension at 72°C for 90 seconds and final elongation at 72° C for 10 minutes. Glyceraldehyde-3-phosphate dehydrogenase (GAPDH) was used as a housekeeping gene to show the integrity of the RNA and the assay. Specific primer pairs were designed from the cDNA sequences available at Gen Bank [27] using Primer Express software (Applied Biosystems) and ordered from Eurogentec, B-4102 Serain, Belgium. For primer sequences see Table 1. The PCR products were separated on a 2% agarose gel stained with ethidium bromide and detected using Molecular Image Gel Doc XR (Biorad, Reinach, CH).

**Table 1:** Sequences for primers and TaqMan probes

Primer	Primer Sequence	Product length	Accession #
RT-PCR			

VDR forward VDR reverse	5'-TGGTGACTTTGACCGGAATGT-3' 5'-CGCCTGAAGAAACCTTTGCA-3'	111 bp	NM 001163959.1

TRPV5 forward TRPV5 reverse	5'-CCCGAGATAACACTATCCTC-3' 5'-CAGGAAGGCATAGGTGATGA-3'	221 bp	AY944068.1

TRPV6 forward TRPV6 reverse	5'-AACTTGTCTTTGAGCCCATGACA-3' 5'-TTCACATTCTGATTCATAATGGCTACAT-3'	76 bp	XM 001490905

CB forward CB reverse	5'-TTTACTGAAAGGTTCAAGCTCCATT-3' 5'-AACACCTGGAATTCTTCAAAACTAACTT-3'	98 bp	AY229893.1

GAPDH forward GAPDH reverse	5'-AAGGGTCATCATCTCTGCTCCTT-3' 5 '-TGACAATCTTCAGGGAATTGTCA-3'	91bp	NM 001163856.1

Real-time TaqMan PCR			

VDR forward VDR reverse VDR probe	5'-TGGTGACTTTGACCGGAATGT-3' 5'-CGCCTGAAGAAACCTTTGCA-3' 5'-AGACCGAGCCACAGGCTTTCATTTCA-3'	111 bp	NM_001163959.1

TRPV6 forward TRPV6 reverse TRPV6 probe	5'-AACTTGTCTTTGAGCCCATGACA-3' 5'-TTCACATTCTGATTCATAATGGCTACAT-3' 5'-CTGAGCTCTTTGAGGGTCAAACTGCATTG-3'	76 bp	XM 001490905

CB forward CB reverse CB probe	5'-TTTACTGAAAGGTTCAAGCTCCATT-3' 5'-AACACCTGGAATTCTTCAAAACTAACTT-3' 5'-TCAAAGAACTGGACAAGAACGGAGATGGA-3'	98 bp	AY229893.1

β-actin forward β-actin reverse β-actin probe	5'-TCATCACCATCGGCAACGA-3' 5'-CGTCACACTTCATGATGGAGTTGA-3' 5'-ATGGAATCCTGTGGCATCCACGAAACTAC-3'	102 bp	NM 001081838.1

### Quantitative Real-Time (TaqMan) PCR

The VDR, TRPV6 and CB mRNA expression levels were quantified in the different intestinal sections by quantitative real-time (TaqMan) PCR using the ABI 7500 Fast Real-Time PCR System (Applied Biosystems, Rotkreuz, CH). Samples were run in duplicates in a 25-μl reaction mixture containing 12.5 μl FastStart Universal Probe Master (Roche), 300 nM of each primer, 200 nM TaqMan probe and 5 μl cDNA. Autoclaved water instead of RNA and the so-called RT minus control were used as negative controls, assuring that there was no contamination of the reagents used for the reactions and confirming the accuracy of the DNase treatment. The primers as well as the 6-carboxyfluorescein (6-FAM)- and 6-carboxytetramethyl-rhodamine (TAMRA)-labelled probes were designed from the cDNA sequences available at Gen Bank [27] using Primer Express Software (Applied Biosystems) and ordered from Eurogentec, B-4102 Serain, Belgium. For primer sequences and TaqMan probe sequences see Table 1. The expression levels of VDR, TRPV6 and CB were evaluated using the comparative CT method (ΔΔ CT method) according to the instructions of the manufacturers of the ABI 7500 Fast Real-Time PCR System; these levels were calculated relative to the expression of the reference gene ß-actin and normalised to the calibrator. The sample with the lowest level of the respective target gene transcripts was used as the calibrator. The efficiency of the PCR reactions was measured using the CT slope method with a range of 10-fold serial dilutions of the targeted PCR product and using samples from different segments of the intestine (for testing the efficiency at different quantities of the targeted RNA expected in different segments) according to the instructions of the manufacturer of the ABI 7500 Fast Real-Time PCR System. The assay was established to assure approximately 100% efficiency of reactions. The specificity of the selected PCR products was confirmed by sequencing (Microsynths, Switzerland).

### Western Blot

The tissue samples of all intestinal segments and animals were homogenised in NET-2 buffer (50 mM TRIS-HCl, pH 7.4, 300 mM NaCl, 0.05% Nonidet P-40 [28]) containing 1 μl/ml protease inhibitor (Sigma-Aldrich, Buchs, CH). After centrifugation (1000 *g*, 10 minutes, 4°C), the clarified supernatants were removed, and the protein concentrations were determined by the Bradford method (Bradford, 1976) (Bio-Rad Laboratories, Reinach, CH). According to their concentrations, the samples were diluted with NET-2 buffer and sample buffer, which contained β-mercaptoethanol, and incubated for 5 minutes at 95°C.

Proteins were separated on SDS-Gel (VDR: 8% polyacrylamide, TRPV6: 10% polyacrylamide, CB: 17% polyacrylamide) and transferred onto PVDF membranes. The membranes were blocked in 5% fat free milk in PBS-T (Tris-buffered saline with 0.025% Tween-20) for one hour, followed by an overnight incubation at 4°C with the primary antibody diluted in 2.5% milk/PBS-T. The antibodies were: a rat monoclonal anti-VDR antibody (ab8756, Abcam, Cambridge, UK) and a rabbit polyclonal anti-CB antibody (Swant, Bellinzona, CH). The detection of TRPV6 protein was not possible due to the lack of a specific antibody suitable for western blot. The following antibodies were tested: (i) rabbit polyclonal anti-TRPV6 antibody (ACC-036, Alomone Labs, Israel); (ii) rabbit polyclonal anti-TRPV6 (H-90, sc-28763, Santa Cruz Biotechnology, Santa Cruz, CA, USA); (iii) rabbit-polyclonal anti-TRPV6 (429); (iv; v) mouse-monoclonal anti-TRPV6 (20C6 and 26B3). The last three antibodies (iii, iv, v) were kindly provided by Prof. Dr. Veit Flockerzi, Experimentelle und Klinische Pharmakologie und Toxikologie, Universität des Saarlandes, D-66421, Homburg, Germany.

Following incubation, the blots were washed three times for 10 minutes in PBS-T and incubated with the secondary peroxidase-conjugated antibody for one hour at room temperature (rabbit polyclonal anti-rat-H&L (HRP), ab6734, Abcam, Cambridge, UK; anti-rabbit IgG (H+L) HRP conjugate, Promega, Dübendorf, CH). After washing the membranes five times in PBS-T for 10 minutes, the protein detection was performed by chemiluminescence (Immun-Star HRP Substrate Kit, Bio-Rad) and exposed to LAS-3000 (Fujifilm).

The membranes were reblotted with anti-β-actin antibody as the loading control. The optical density of the VDR, CB and β-actin bands was quantified using ImageJ software. The values are presented as the ratio of VDR and CB optical density to the corresponding β-actin optical density.

### Immunohistochemical analysis

Sections (3 μm) of the formalin-fixed and paraffin-embedded tissue samples were dewaxed using xylene, rehydrated through serial dilutions of ethanol to water and rinsed in Trizma-buffered saline for 2 minutes. Afterwards, the antigen retrieval was performed by boiling the sections in citrate buffer in a microwave oven (600 W, 3 × 5 min) and rinsing in Tris-buffered saline (TBS, buffer stock solution: 6.1 g trizma base, 50 ml H2O and 37 ml 1 N HCl, diluted with H_2_O to 1000 ml solution, pH adjusted to 7.6; working solution: 100 ml buffer stock solution plus 900 ml saline, 0.85%).

To block the endogenous peroxidase activity, the sections were incubated in 0.3% H_2_O_2 _in methanol for 15 minutes. Non-specific binding was blocked with Protein-Block-Serum-free (VDR, Dako Schweiz AG, Baar, CH) or 10% normal goat serum (CB). The tissue sections were incubated overnight with the primary antibody. The expression of VDR was detected with a rat monoclonal anti VDR antibody (ab8756, Abcam, Cambridge, UK), which reacts with the C-terminal of the DNA-binding domain with occupied and unoccupied forms of the VDR. The expression of CB was detected with a rabbit polyclonal anti-CB-antibody (Swant, Bellinzona, CH), which was produced against rat recombinant Calbindin-D9k protein. As a negative control, the tissue sections were incubated with TBS instead of the primary antibody. After rinsing in TBS for 3x10 minutes, the biotinylated secondary antibodies were added for a 30-min incubation at room temperature (VDR: anti-rat-IgG, EO468, Dako, Glostrup, DK; CB: PowerVision Poly-HRP IHC Detection System, anti-Rabbit IgG). After washing in TBS for 2x10 minutes, the tissue sections were incubated for 30 minutes with StreptABComplex/HRP Duett (Dako, Glostrup, DK), and Chromogen diaminobenzidine tetrahydrochloride (Liquid DAB+ Substrate, Dako, Baar, CH) was added. Before being automatically coverslipped (RCM 2000^®^; Medite) in Pertex^® ^(Medite), the sections were counterstained with haematoxylin.

### Ussing Chamber Technique

The DD, CC, CAD, and CD tissue specimens were mounted in a modified Ussing chamber [29] and bathed with 3.5 ml of Parson buffer solution on both sides of the intestinal wall. The epithelium was continuously short-circuited by an automatic voltage-clamp device (Aachen Microclamp, AC Copy Datentechnik, Aachen, Germany) with correction for solution resistance. The tissue conductance (G_t_) was measured by recording the voltage resulting from the bipolar current pulses (± 100 mA) applied across the tissue at 1-minute intervals and calculated according to Ohm's law. The values for G_t _and the continuously applied short-circuit current (I_sc_) were recorded each minute. Ten minutes after mounting the tissues in the chambers, 10 μl ^45^Ca^2+ ^was added to one side of the intestinal wall (the labelled side). After an additional incubation of 60 minutes to allow the isotope flux rates to reach a steady state and I_sc _to stabilise, the unidirectional ion flux rates were determined in sequential 20-minute periods. From the measured unidirectional net flux rates (J_ms _= flux rate from mucosa to serosa; J_sm _= flux rate from serosa to mucosa), the net ion flux rates (J_net_) were calculated according to J_net_ = J_ms _- J_sm _(nmol/h/cm^2^) from the mean unidirectional flux rates.

### Statistics

The data sets of real-time and western blot were tested for normality using the Kolmogorov-Smirnov Test. Small deviations from normality were observed in some data sets. Thus, the data were analysed by a nonparametric one-way analysis of variance using the Kruskal-Wallis test, followed by Dunnett's multiple comparison test. For all of the statistical analyses, the software programme GraphPad Prism 5.0 (GraphPad Software Inc., San Diego, Ca, USA) was used. P < 0.05 was considered statistically significant. Data are presented as means ± SD.

## Results

### Expression of VDR, CB, TRPV6 and TRPV5 mRNA in the horse intestine

**Figure 1:**
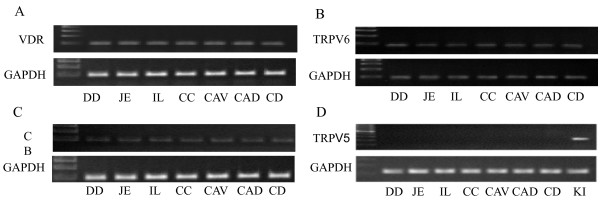
**Reverse transcriptase PCR**. Detection of VDR, calbindin-D9k (CB), TRPV6 and TRPV5 cDNA in different intestinal segments (DD = duodenum, JE = jejunum, IL = ileum, CC = cecum, CAV = colon ascendens ventrale, CAD = colon ascendens dorsale, CD = colon descendens) detected by reverse transcriptase PCR. GAPDH was used as a loading control. VDR, TRPV6 and CB cDNA was found in all investigated segments (Figure 1A, B, C), while TRPV5 was solely detected in the kidney (KI), not in the horse intestine (Figure 1D).

The amplified VDR, TRPV6 and CB cDNA with the expected sizes was detected in all of the intestinal segments and was investigated by qualitative reverse transcriptase PCR (Figure 1A, B, C). TRPV5 cDNA expression was not detectable in the intestine (Figure 1D).

**Figure 2:**
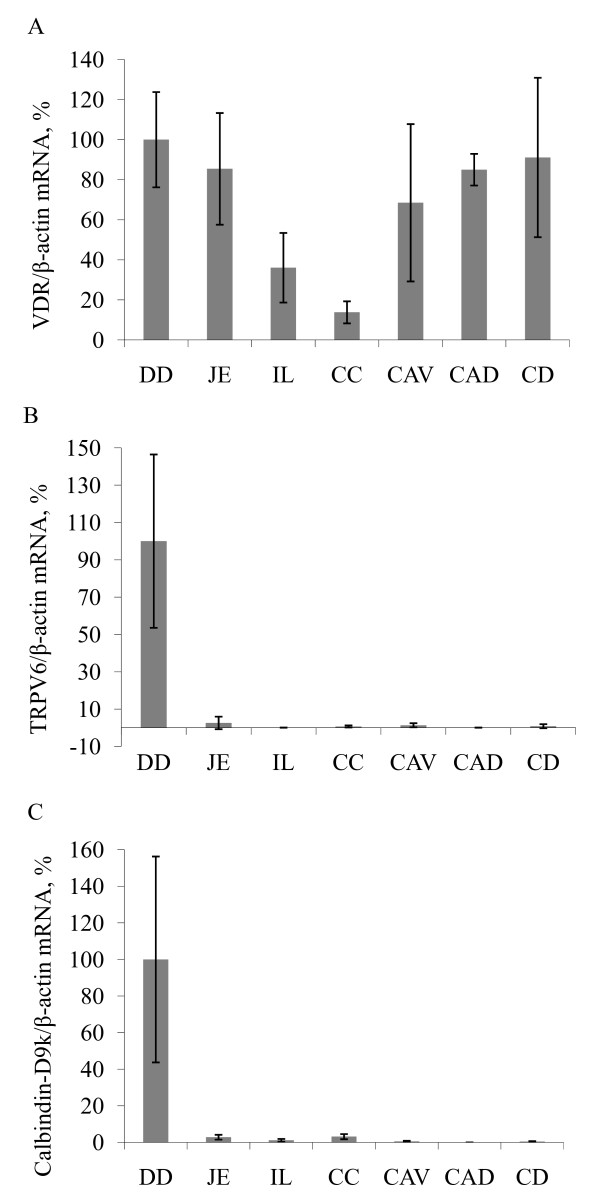
**Real-time PCR**. VDR, CB, and TRPV6 mRNA expression levels in the different intestinal segments in the horse intestine (DD = duodenum, JE = jejunum, IL = ileum, CC = cecum, CAV = colon ascendens ventrale, CAD = colon ascendens dorsale, CD = colon descendens) were determined by real-time (TaqMan) PCR. Expression was normalised to the expression of β-actin. VDR mRNA was found in each intestinal segment, but the amount and distribution patterns showed distinct inter-individual variations between the animals investigated. There were no significant differences between VDR mRNA expression levels in the small and large intestine (p > 0.05) (Figure 2A). The duodenum expressed the highest levels of TRPV6 and CB mRNAs (p < 0.05) (Figure 2B, C).

VDR mRNA was found in each intestinal segment, but the amount and distribution patterns showed distinct inter-individual variations between the animals investigated. There were no significant differences between the VDR mRNA expression levels in the small and large intestine (p > 0.05) (Figure 2A).

The highest levels of TRPV6 and CB mRNA were detected in the duodenum. The mRNA content in the remaining segments was significantly lower compared with the duodenum (p < 0.05) (Figure 2B, C).

### Expression of VDR and CB protein in different intestinal segments

**Figure 3:**
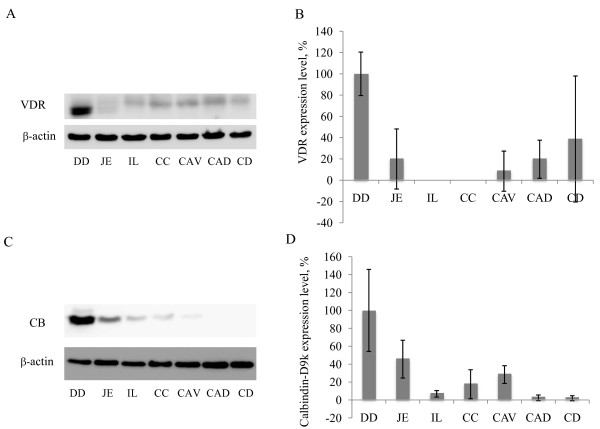
**Western blot analyses**. Expression of VDR and CB proteins in the horse intestine was determined by western blot analysis. Tissue samples of the duodenum (DD), jejunum (JE), ileum (IL), cecum (CC), colon ascendens ventrale (CAV), colon ascendens dorsale (CAD) and colon descendens (CD) were used. The loading was controlled by ß-actin. A) A representative western blot of VDR protein expression is shown. In all of the animals, major bands of approximately 40 kDa were found in the DD, while minor bands were detectable in the jejunum and colon in three animals. B) Relative expression of VDR to ß-actin was determined by densitometry using ImageJ software. The highest expression was measured in the DD. C) The strongest bands of CB protein were detected in the duodenum in all of the animals. Minor bands were visible in the jejunum. In 9 animals, weak signals were found in cecum, and in 6 animals, weak bands were seen in the ileum and colon ascendens ventrale. D) The expression of CB protein relative to ß-actin was determined by measuring the optical density using ImageJ software. The highest CB protein expression was found in the DD.

Prominent VDR protein bands of approximately 50 kDa were detectable in the duodenum of all the animals investigated by western blot analyses (Figure 3A). Additionally, significantly weaker VDR protein bands were observed in the jejunum, the colon ascendens dorsale and the colon descendens in three animals, and in one animal, a minor band was found in the colon ascendens ventrale (Figure 3B)

The strongest CB protein bands of approximately 9 kDa were detected in the duodenum. Minor bands were observed in the jejunum in all of the animals; 9 animals had low expression in the cecum, and in 6 horses, weak bands were visible in the ileum and the colon ascendens ventrale (Figure 3C, D).

### Localisation of VDR and CB protein in the horse intestine

**Figure 4:**
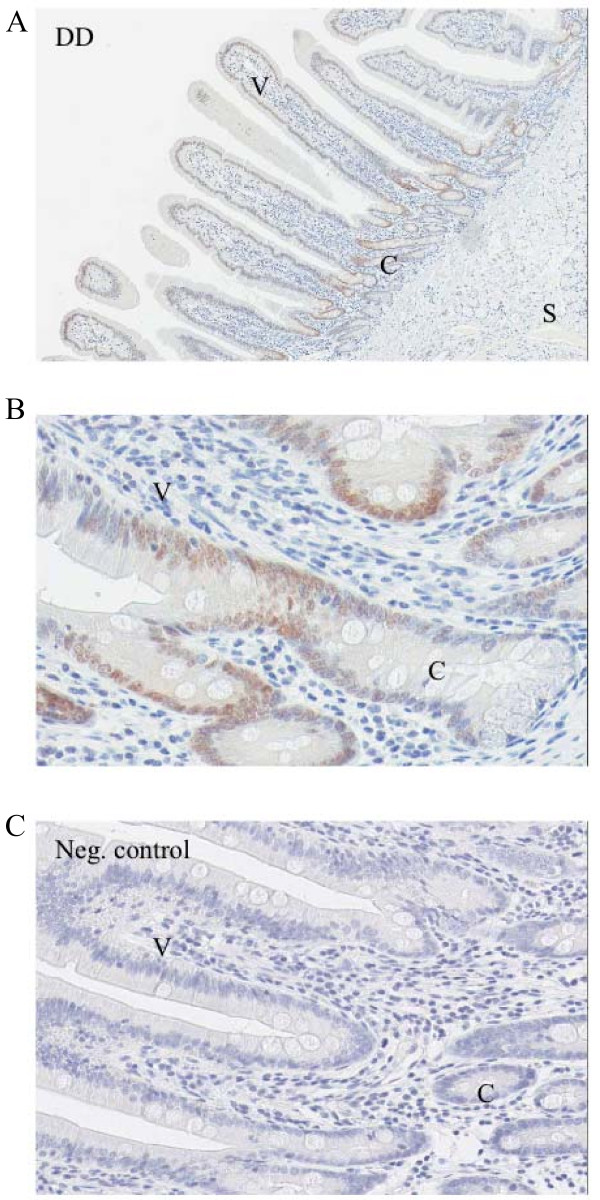
**Immunohistochemistry VDR**. Immunohistochemical analyses were performed to localise the VDR protein in the horse intestine. A) VDR protein was mainly detected in the superficial crypts of the duodenum. B) Tissue from Figure 4A at a higher magnification. Inside the enterocyte, the strongest labelling was found in the nuclei, while weaker signals were seen in the cytoplasm. C) Negative control. V = villus, C = crypt, S = submucosa.

**Figure 5:**
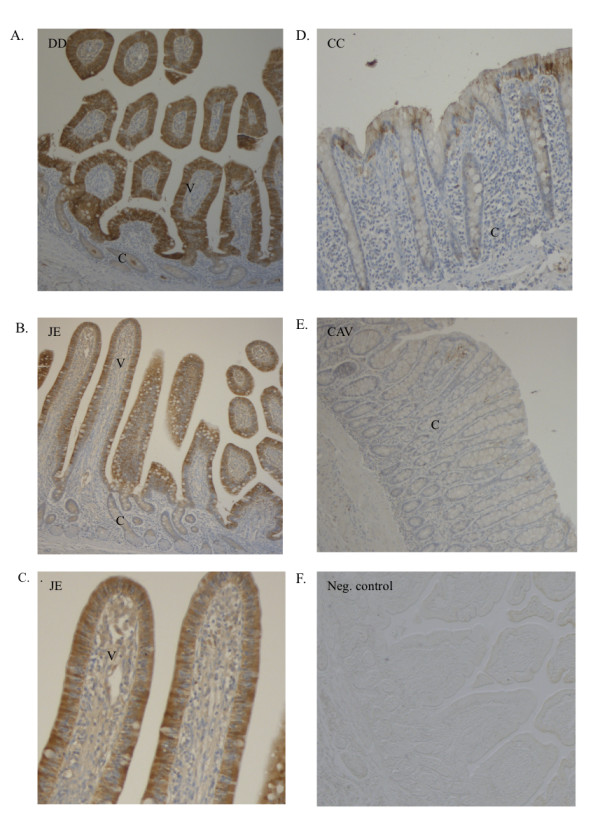
**Immunohistochemistry Calbindin-D9k**. Immunohistochemistry was performed to localise calbindin-D9k expression in the intestinal wall. The strongest labelling was visible in the villi of the duodenum (DD) followed by that of the jejunum (JE). Considerably weaker staining was observed in the cecum (CC) and in the colon ascendens ventrale (CAV). Inside the enterocytes, the strongest staining was seen in the cytoplasm.

To determine the localisation of VDR and CB protein in the intestinal wall, immunohistochemistry was performed (Figure 4, 5). VDR immunolabelling revealed staining mainly in the nuclei of the superficial crypts of the duodenal enterocytes (Figure 4A). In the enterocyte, the strongest staining was observed in the nuclei, while the weaker signals were found in the cytoplasm (Figure 4B).

The strongest labelling of CB was seen in the villi of the duodenum, followed by the jejunum. The staining in the cecum and in the colon ascendens ventrale was weaker. In the enterocyte, the strongest staining was visible in the cytoplasm.

### Ex-vivo Ca^2+ ^absorption

**Figure 6:**
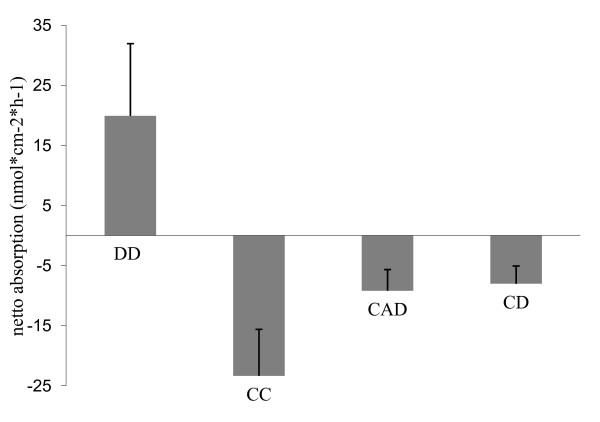
**Ussing chamber**. Ex-vivo Ca^2+ ^absorption in different intestinal segments (duodenum = DD, cecum = CC, colon descendens dorsale = CAD, colon descendens = CD) was measured by the Ussing chamber technique. Positive flux rates (absorption) from the mucosal to the serosal sides were measured in the duodenum, while in the remaining segments (CC, CAD, CD), the negative flux rates (secretion) were determined.

The ex-vivo Ca^2+ ^absorption was measured using the Ussing chamber technique. The net Ca^2+ ^flux rates revealed high inter-individual variability in all of the tested segments. The mucosal to serosal Ca^2+ ^flux rates (Jms) exceeded the flux rates in the opposite direction (Jsm) only in the duodenum. This resulted in net Ca^2+ ^flux rates (Jnet = Jms-Jsm) ranging between 12.2-54.3 nmol/cm^2^h^-1 ^in the duodenum. The other investigated segments the cecum, colon ascendens, and colon descendens showed negative flux rates, so Jsm exceeded Jms. The mean Ca^2+ ^flux rates in the hindgut ranged between -8 in the colon ascendens and -23.3 nmol/cm^2^h^-1 ^in the cecum. Ca^2+ ^absorption was only observed in the duodenum (Figure 6).

## Discussion

The current study was performed to examine the expression patterns of the Ca^2+ ^transport elements VDR, CB and TRPV6 in the horse intestine and further investigate the ex-vivo Ca^2+ ^absorption via the Ussing chamber technique.

It is well documented that the small intestine is the main site of active intestinal Ca^2+ ^absorption in many species [4,15,18]; however, marked variations between different species have been observed [17,30-32].

With regard to the Ca^2+ ^metabolism, horses show differences compared with most monogastric animals because they have remarkably high serum Ca^2+ ^concentrations, low mean serum calcidiol and vitamin D concentrations, high intestinal Ca^2+ ^absorption; they depend on the Ca^2+ ^content in their feed, and excess Ca^2+ ^is eliminated via the urine [18,19,24,25,33].

These physiological characteristics are similar to those of other hindgut fermenters such as rabbits and other small wild herbivores. Because the rabbit, a cecum fermenter, absorbs a significant amount of Ca^2+ ^in the cecum [22,23,26], the question arises if the horse, as a hindgut fermenter, also shows peculiarities concerning the site of active intestinal Ca^2+ ^absorption.

Therefore, the intestinal protein and mRNA expression of the Ca^2+ ^transport elements VDR, TRPV6, and CB were investigated, and the active Ca^2+ ^absorption was measured ex-vivo in different intestinal segments by the Ussing chamber technique.

High mRNA levels of TRPV6 and CB as well as high protein levels of VDR and CB in the duodenum indicated that this is the main site of Ca^2+ ^absorption in the horse. These observations were further supported by the results of the Ussing chamber technique, demonstrating that the only measurable directed ex vivo Ca^2+ ^transport through intestinal epithelium i.e. from mucosal to serosal side was found in the duodenum. These findings confirmed the idea that transcellular movements occur mainly in segments where the sojourn time of the chyme is very short so that a maximum amount of Ca^2+ ^could be absorbed during this short period [34]. Similar results have been documented in a Ussing chamber study performed by Cehak et al. [35], where highest ex-vivo Ca^2+ ^absorption were measured in the duodenum of horses, followed in descending order by the jejunum, colon ascendens dorsale and cecum. In the present study, the duodenum was the only segment where the absorbance was measurable, while secretion of Ca^2+ ^was recorded in the remaining investigated segments. The extent of active Ca^2+ ^absorption is closely related to feeding [33,36], and thus, the different measurements in the hindgut may be explained through the different Ca^2+ ^content in the feed, particularly as reflected in our results. In addition to the VDR protein expression in the duodenum, protein bands were detectable in the hindgut in three animals. This result may indicate that active Ca^2+ ^absorption occurs in the hindgut, and this absorption may be activated if dietary Ca^2+ ^content is reduced or demand increased. This idea is supported by the detection of CB and TRPV6 mRNA in the hindgut, although the expression levels were low. The regulation of VDR has been reported in the hindgut of ruminants. Goff et al. [37] observed that VDR expression in Jersey cows was 3-4 fold higher during late pregnancy than in non-lactating cows. A significant decrease of VDR expression in the colon is described post-partum in sheep and goats by Liesegang et al. [38]. Therefore, it is conceivable that the inter-individual variations between the animals used for this study are due to different physiological status or previous keeping and feeding of the animals. The information about the horses used for this study was limited to age and sex, so further studies with special feeding groups are recommended.

The localisation of VDR in the nuclei, particularly in the crypt, is discussed and observed in goats, cows and rabbits [23,26,39]. Compared with the expression of CB, which is primarily in the adult enterocytes, VDR is mainly expressed in younger cells.

Recently, Rourke et al. [19] measured the mRNA levels of TRPV5/6, CB, VDR, PMCA1 and NCX1 in the horse intestine. In contrast to our study, Rourke et al. [19] found higher levels of TRPV6 and CB mRNA in the proximal jejunum than in the duodenum. This divergence may be the result of different sampling because the samples for this study were taken from the middle part of the jejunum. Thus, the conclusion is restricted to the parts of the intestine investigated in this study, and further research on Ca^2+ ^absorption and protein expression in the proximal jejunum are recommended.

Interestingly, a divergence in the expression of VDR protein and mRNA was observed in this study. The missing correlation resembles the phenomenon described by Mohri et al. [40], which found that human VDR is post-transcriptionally regulated by microRNAs that suppress the translation or degradation of VDR mRNAs. Based on the low VDR mRNA expression in the small intestine, Rourke et al. [19] argued that the transcellular epithelial Ca^2+ ^transport in the horse is not as dependent on VD as in other species. The high levels of VDR proteins in the duodenum in the horses in this study suggested that this interpretation is not accurate and that posttranscriptional regulation of VDR mRNA may play an important role for intestinal Ca^2+ ^absorption in the horse. Thus, VD may be involved in regulatory processes of active Ca^2+ ^absorption in the horse intestine through VDR.

In contrast to Rourke et al. [19], which detected a low level of TRPV5 mRNA in the intestinal tract, we observed no signal in the samples in the present investigation, possibly because the amount of TRPV5 mRNA was under the detection limit.

As described for other mammals, TRPV5 may be the major isoform found in the kidney, whereas TRPV6 expression is primarily detectable in the intestine [41].

## Conclusion

The expression patterns of VDR, TRPV6 and CB indicate that the established three-step mechanism of active Ca^2+ ^absorption occurs in the horse intestine. TRPV5 possibly may not have a major function in this mechanism.

The coincidental presence of TRPV6, CB and VDR (proteins) in the duodenum suggests that active Ca^2+ ^transport occurs predominantly in this segment; these results were further supported by ex-vivo absorption studies performed by Ussing chamber techniques. The expression of VDR protein in the duodenum indicated that VD has a regulatory function in the active intestinal Ca^2+ ^absorption in the horse.

## Authors' contributions

NS was involved in the coordination and performance of most experiments, in the evaluation and interpretation of the data and the drafting of the manuscript. TM was involved in the sampling procedure, in the coordination and performance of most experiments and in the evaluation and interpretation of the data. MPK supervised the performance of the experiments. AL designed the project, was involved in the sampling procedure and supervised the Ussing chamber experiment. AB designed and supervised the project and was involved in drafting the manuscript. All authors read and approved the final manuscript.
